# SIRT1 regulates O-GlcNAcylation of tau through OGT

**DOI:** 10.18632/aging.103062

**Published:** 2020-04-20

**Authors:** Shu Lu, Xiaomin Yin, Jia Wang, Qun Gu, Qin Huang, Nana Jin, Dandan Chu, Ziqi Xu, Fei Liu, Wei Qian

**Affiliations:** 1 Department of Biochemistry and Molecular Biology, Medical School, Nantong, Jiangsu, P. R. China; 2 Jiangsu Key Laboratory of Neuroregeneration of Jiangsu and Ministry of Education of China, Co-innovation Center of Neuroregeneration, Nantong University, Nantong, Jiangsu, P. R. China; 3 Department of Intensive Care Unit, The Affiliated Hospital of Nantong University, Nantong, Jiangsu, P. R. China; 4 Department of Neurochemistry, New York State Institute for Basic Research in Developmental Disabilities, Staten Island, NY 10314, USA

**Keywords:** SIRT1, OGT, O-GlcNAcylation of tau, phosphorylation of tau, Alzheimer’s disease

## Abstract

Tau is modified with O-GlcNAcylation extensively in human brain. The O-GlcNAcylation levels of tau are decreased in Alzheimer’s disease (AD) brain. Sirtuin type 1 (SIRT1) is an enzyme that deacetylates proteins including transcriptional factors and associates with neurodegenerative diseases, such as AD. Aberrant SIRT1 expression levels in AD brain is in parallel with the accumulation of tau. cAMP response element binding protein (CREB), a cellular transcription factor, plays a critical role in learning and memory. In this present study, we found SIRT1 deacetylates CREB and inhibits phosphorylation of CREB at Ser133. The inactivated CREB suppresses OGT expression and therefore decreases the O-GlcNAcylation of tau and thus increases the phosphorylation of tau at specific sites. These findings suggest that SIRT1 may be a potential therapeutic target for treating tauopathies.

## INTRODUCTION

Tau is encoded by the microtubule-associated protein tau (*MAPT*) gene located on the long arm of human chromosome 17 [1]. Tau contributes to tubulin assembly and stabilization of microtubules, thereby regulating normal functions of neurons [2].

Tau is a phosphorylated protein which contains 85 potential serine (S), threonine (T), tyrosine (Y) phosphorylation sites [3]. Normal tau contains 2-3 phosphate moles of phosphate per mol of tau, whereas hyperphosphorylated tau contains 9–10 moles of phosphate [4]. Abnormal hyperphosphorylated tau protein forms intracellular filamentous deposits, therefore resulting in a group of dementias termed tauopathies, such as Alzheimer’s disease (AD), frontotemporal dementia and parkinsonism linked to chromosome 17 (FTDP-17), Pick’s disease (PiD), progressive supranuclear palsy (PSP), corticobasal degeneration, argyrophilic grain disease, globular glial tauopathy, and chronic traumatic encephalopathy [5].

O-GlcNAcylation, a dynamic posttranslational modification (PTM), is a single N-acetylglucosamine (GlcNAc) attached to serine and threonine residues of nuclear and cytoplasmic proteins [6]. Human brain tau is modified with O-linked N-acetylglucosamine (O-GlcNAc) [7]. A marked decrease of O-GlcNAcylation of tau protein has been showed in human AD brain [8]. The down-regulation of tau O-GlcNAcylation in AD can cause tau pathology and neurodegeneration [9]. The “Yin-Yang” reciprocal relationships between O-GlcNAcylation and phosphorylation have been suggested since phosphorylation and O-GlcNAcylation occur on the same Ser/Thr site of protein [10]. It has been shown that O-GlcNAcylation negatively regulates tau phosphorylation in a site-specific manner in cultured cells, in vivo and in metabolically active brain slices [11, 12]. Tau-mediated neurodegeneration may be promoted by decreased O-GlcNAcylation through abnormal hyperphosphorylation and oligomerization of tau [9]. The only processing enzymes of O-GlcNAcylation are O-GlcNAc transferase (OGT) and O-GlcNAcase (OGA) [7]. OGT catalyzes the addition of N-acetylglucosamine to serine or threonine hydroxyl groups of target proteins [13]. OGA removes O-GlcNAc from proteins [14–16].

CREB is a cAMP-responsive transcription factor regulating the somatostatin gene [17]. CREB binds to the cAMP response element (CRE) in the promoters of its target genes, upon phosphorylation at Ser133 by different protein kinases, such as protein kinase A (PKA), calmodulin-dependent protein kinase (CaMK), mitogen-activated protein kinases (MAPK), and so on [18]. Transcription of memory-associated genes is associated with CREB phosphorylation, disruption of which in AD results in memory impairment [19]. Deacetylation prevents cAMP-dependent phosphorylation of CREB to affect its activity [20].

Sirtuins (SIRT1–7) were first identified in yeast and belong to class III histone deacetylases (HDACs) [21]. SIRT1 is the ortholog of the yeast Sir2 and the most evolutionally conserved member [22]. SIRT1 has neuroprotective properties in an array of neurological disorders, such as AD, Parkinson’s disease, and Huntington’s disease [23]. It is well known that SIRT1 regulates energy (glucose and lipid) metabolism in peripheral tissues [24]. SIRT1 can deacetylate a large number of transcription factors including the forkhead box class O (FoxO) family members [25, 26], P53 and nuclear factor κB (NF-κB) [27, 28]. SIRT1 also deacetylates CREB at Lys136 to regulate its activity [20].

Whether and how SIRT1 regulates O-GlcNAcylation and phosphorylation of tau remain elusive. In the present study, we investigated the role of SIRT1 in OGT expression. We found that SIRT1 deacetylated CREB to suppress OGT expression and therefore to decrease the O-GlcNAcylation of tau and increase the phosphorylation of tau at specific sites.

## RESULTS

### SIRT1 reduces O-GlcNAcylation modification of tau.

**Figure 1:**
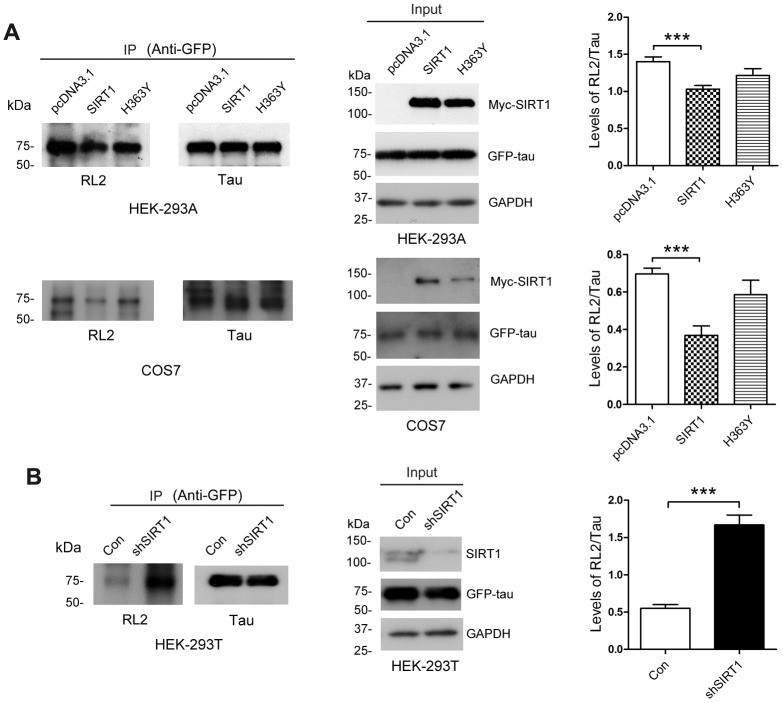
**SIRT1 decreases protein O-GlcNAcylation of tau in cells.** (**A**) pcDNA3.1, pcDNA3.1/Myc-SIRT1 or pcDNA3.1/Myc-H363Y was transfected into GFP-Tau_441_ overexpressed HEK-293A or COS7 cells. Recombinant human tau_441_ immunoaffinity-purified with anti-GFP antibody from HEK-293A cells was immunolabeled with anti-GFP to tau, RL2 to O-GlcNAc or anti-Myc to SIRT1. Quantitative analysis of relative O-GlcNAcylation levels of tau after being normalized with total tau is shown as mean ± S.D. (n=3), ***, *p* < 0.001; (**B**) shSIRT1 plasmids or its control plasmids were transfected into HEK-293T cells. Recombinant human tau_441_ was immunoaffinity-purified with anti-GFP antibody from cells. Quantitative analysis of relative O-GlcNAcylation levels of tau after being normalized with total tau is shown as mean ± S.D. (n=3), ***, *p* < 0.001.

SIRT1 influences glucose metabolism [24]. To investigate whether the O-GlcNAcylation level of tau is regulated by SIRT1, GFP-Tau_441_ was co-overexpressed together with Myc-SIRT1 or Myc-H363Y, the deacetylase-dead mutation of SIRT1, in HEK-293A or COS7 cells. After 48 h, we purified GFP-tau protein from cell lysates by immunoprecipitation using anti-GFP antibody. We found that SIRT1 but not H363Y could decrease the O-GlcNAcylation level of tau protein significantly in both cell lines (Figure 1A). In the mean time, we transfected shSIRT1 plasmids into HEK-293T cells to knockdown endogenous SIRT1 level and detected the O-GlcNAcylation level of immunoprecipitated GFP-tau. We found the O-GlcNAcylation level of GFP-tau was significantly increased due to decline of SIRT1 expression (Figure 1B). These data suggested that SIRT1 could reduce O-GlcNAcylation modification of tau in cells.

**Figure 2:**
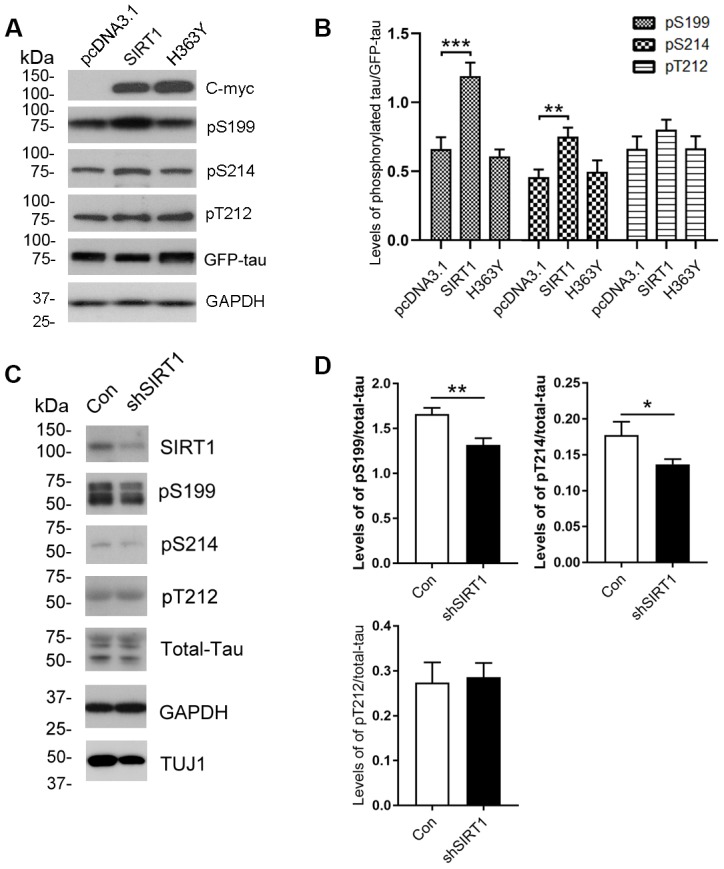
**Changes of site-specific phosphorylation levels of tau in HEK-293A cells and primary cortical neurons.** (**A**) The levels of total tau and the indicated site-specific phosphorylation levels of tau in the extracts of HEK-293A cells transfected with GFP-tau_441_ together with SIRT1 or H363Y were analyzed by western blot developed with anti-GFP antibody and with several phosphorylation-dependent/site-specific tau antibodies shown in right side of the panel. (**B**) Blots in panel A were quantified after normalization with the GFP-tau level, and the relative levels of site-specific tau phosphorylation are shown as mean ± S.D. (n=3), **, *p* < 0.01; ***, *p* < 0.001. (**C**) The levels of total tau and tau phosphorylated at the indicated phosphorylation sites in the extracts of cortical neurons of E18 rats were analyzed by western blots developed with R134d against total tau and with several phosphorylation-dependent/site-specific tau antibodies shown in right side of the panel. Tuj1 was used as a neuronal cell marker for western blot. The cortical neurons of E18 rats were infected with lentiviral-shSIRT1 or its empty vectors for 3 days to knockdown the endogenous expression level of SIRT1. The virus containing empty vectors were used as controls. (**D**) Blots in panel C were quantified after normalization with the total tau level, and the relative levels of site-specific tau phosphorylation are shown as mean ± S.D. (n=3), *, *p* < 0.05, **, *p* < 0.01.

To determine whether the altered O-GlcNAcylation may mediate the phosphorylation of tau, purified tau from HEK-293A cells was immunostained by using antibodies that recognize phosphorylated tau at their specific epitopes. We observed increased tau phosphorylation at Ser199 and Ser214 but not Thr212 due to SIRT1 overexpression (Figure 2A, 2B). However, these changes were not obviously observed in H363Y transfected cells. To further confirm the observations, we determined the phosphorylation levels of endogenous tau in the E18 rat cerebral cortical neurons. We detected the decreased phosphorylation levels of tau at Ser199 and Ser214 by infecting neurons with lentiviral-shSIRT1 (Figure 2C, 2D). These data strongly suggest that the decrease in O-GlcNAcylation of tau protein may be accompanied by hyperphosphorylation of tau at some phosphorylation sites.

### SIRT1 inhibits the expression of OGT

**Figure 3:**
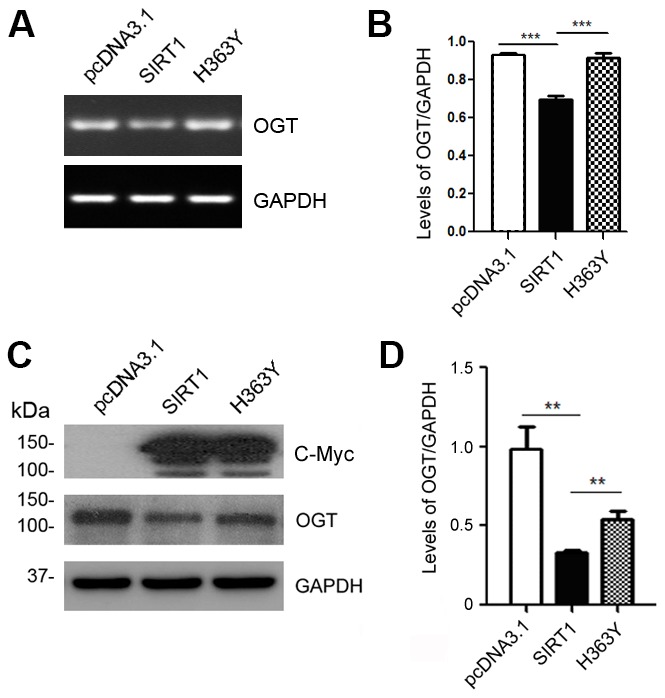
**SIRT1 inhibits OGT expression.** HEK-293A cells were transfected with pcDNA3.1, pcDNA3.1/Myc-SIRT1 or pcDNA3.1/Myc-H363Y. (**A**) mRNA levels of OGT and GAPDH were measured by RT-PCR. (**B**) The quantification of relative mRNA level of OGT after normalization with the mRNA level of GAPDH was represented as mean ± S.D. (n = 3); ***, *p* < 0.001. (**C**) Protein levels of Myc-SIRT1 or Myc-H363Y were analyzed by western blot developed with anti-Myc antibody. GAPDH was used as the loading control. (**D**) Blot shown in panel C was quantified for protein expression levels of OGT after being normalized with GAPDH level. Data are presented as mean ± S.D. (n=3), **, *p* < 0.01.

The O-GlcNAc transferase (OGT) regulates the O-GlcNAc modification on tau proteins [29]. To determine whether SIRT1 controls the mRNA and protein levels of OGT, we transfected HEK-293A cells with pcDNA3.1/Myc-SIRT1 or pcDNA3.1/Myc-H363Y. As expected, SIRT1 reduced the mRNA level of OGT (Figure 3A, 3B). Additionally, we detected the changes of OGT protein levels in HEK-293A cells transfected with SIRT1 or H363Y plasmids. We found the OGT protein level was decreased significantly due to SIRT1 overexpression, whereas the H363Y transfection has little impact (Figure 3C, 3D). These results suggest that SIRT1 may regulate the expression of OGT both at the mRNA and protein levels.

### SIRT1 negatively regulates the expression of luciferase driven by OGT promoter

**Figure 4:**
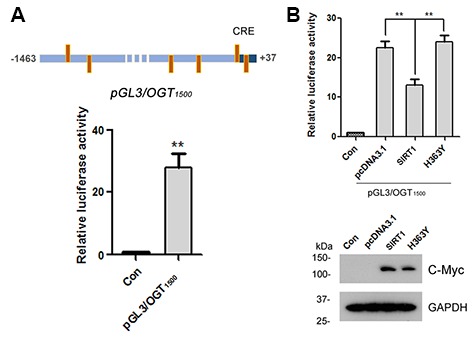
**SIRT1 suppresses luciferase expression driven by OGT promoter.** (**A**) The upper panel represents the schematic diagram of constructed plasmid of pGL3/OGT_1500_, a luciferase reporter plasmid driven by human OGT promoter (−1463 ~ +37). There are six CRE-like elements within human OGT promoter region -1463 ~ +37 bp. pGL3/ OGT_1500_ or pGL3-basic vector was co-transfected together with pRL-Tk into HEK-293A cells. After 48 h transfection, the luciferase activity was measured and normalized with Renilla luciferase (pRL-TK). The relative activity of luciferase was presented as mean ± S.D., **, *p* < 0.01. (**B**) pGL3/ OGT_1500_ or pGL3-basic vector was co-transfected together with pRL-Tk into HEK-293A cells overexpressing Myc-SIRT1 or Myc-H363Y. After 48 h transfection, the luciferase activity was measured and normalized with Renilla luciferase (pRL-TK). The relative activity of luciferase was presented as mean ± S.D., **, *p* < 0.01.

To understand the molecular mechanisms underlying OGT expression regulation, the promoter of the human *OGT* gene was analyzed by MatInspector software analysis [30, 31], a Genomatix internationally renowned program for the identification of transcription factor binding sites. The bioinformatic analysis revealed an array of putative nuclear factor binding sites, especially several potential CRE-like elements (Figure 4A), suggesting CREB may be involved in regulating OGT expression. To investigate the transcriptional regulation of OGT, we inserted the promoter region of human OGT, -1463 ~ +37, into pGL3-basic vector to generate reporter plasmid of pGL3/OGT_1500_. We transfected pGL3/OGT_1500_ together with pRL-TK into HEK-293A cells and then measured luciferase activity using the dual luciferase assay. We found that pGL3/OGT_1500_ increased luciferase activity by almost 30-fold (Figure 4A) compared with pGL3-basic, indicating that the promoter of human OGT drives the luciferase expression. To detect the effect of SIRT1 on OGT transcription, pcDNA3.1, pcDNA3.1/Myc-SIRT1 or pcDNA3.1/Myc-H363Y was co-transfected with pGL3/OGT_1500_ into HEK-293A cells. Cell lysates were analyzed using western blots and luciferase activity assay. We observed a half decrease in luciferase activity in pcDNA3.1/Myc-SIRT1 transfected HEK-293A cells compared with the control transfected cells (Figure 4B), suggesting that overexpression of SIRT1 but not H363Y inhibited the expression of luciferase driven by OGT promoter. These results indicate that SIRT1 may suppress OGT transcription.

### CREB takes part in the expression regulation of OGT

**Figure 5:**
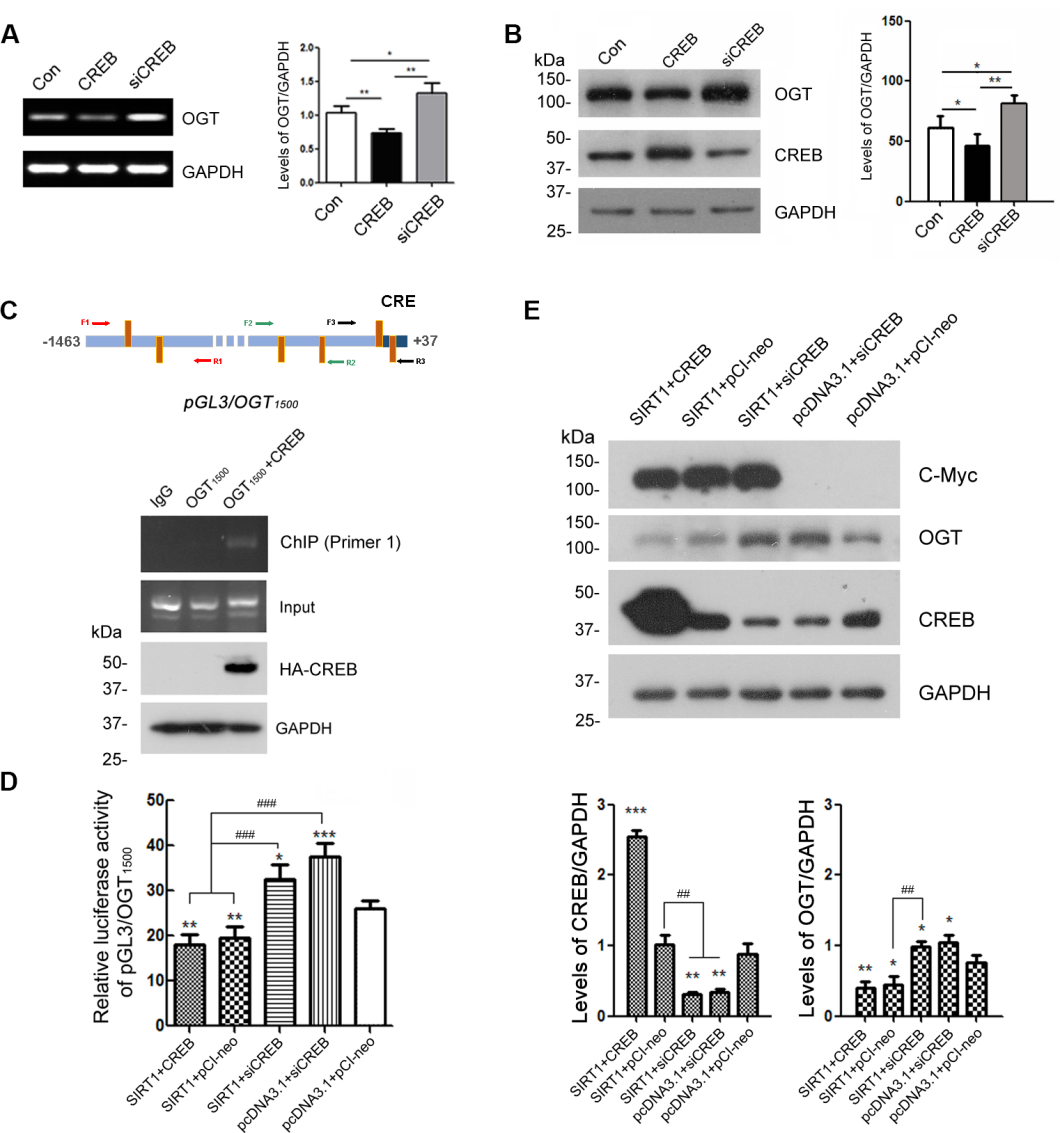
**CREB is involved in the regulation of OGT expression.** HEK-293A cells were transfected with pCI-neo, pCI/HA-CREB or siRNA of CREB respectively. (**A**) mRNA levels of OGT and GAPDH were measured by RT-PCR. The quantification of relative mRNA level of OGT after normalization with the mRNA level of GAPDH was represented as mean ± S.D. (n = 3); *, *p* < 0.05, **, *p* < 0.01. (**B**) Protein levels of OGT, CREB and GAPDH were examined by western blot using anti-OGT, anti-CREB and anti-GAPDH antibody. Relative OGT or CREB level was quantified after normalization with the protein level of GAPDH and presented as mean ± S.D. (n=3), *, *p* < 0.05; **, *p* < 0.01. (**C**) pCI/HA-CREB was co-transfected with pGL3/OGT_1500_ into HEK-293T cells. HA-CREB was immunoprecipitated with anti-HA antibody. Co-immunoprecipitated DNA of OGT promoter with CREB was determined by PCR with three sets of primers specific to CRE elements as indicated for amplifying the DNA. The PCR product was separated by agarose electrophoresis. (**D**) In HEK-293A cells transfected with pGL3/ OGT_1500_ and pRL-TK, pCI-neo, pCI/HA-CREB or siRNA of CREB was transfected with or without overexpression of SIRT1. After 48 h transfection, the luciferase activity was measured and normalized with Renilla luciferase. The relative activity of luciferase was presented as mean ± S.D. (n=3), *, *p* < 0.05, **, *p* < 0.01, ***, *p*< 0.001 versus control group; ###, *p*< 0.001. (**E**) pCI-neo, pCI/HA-CREB or siRNA of CREB was transfected into HEK-293A cells overexpressing SIRT1 or not. Protein levels of SIRT1, OGT, CREB or GAPDH were detected by western blot using anti-Myc, anti-OGT, anti-CREB or anti-GAPDH antibody. Relative OGT or CREB level was quantified after normalization with the protein level of GAPDH and presented as mean ± S.D. (n=3), *, *p* < 0.05; **, *p* < 0.01; ***, *p* < 0.001 versus control group; ##, *p*< 0.01.

Several CRE-like elements appeared densely in the OGT promoter according to MatInspector software analysis (Figure 4A). Therefore, we want to explore whether CREB participates in the regulation of OGT expression. We overexpressed CREB or knocked down CREB by siRNA of CREB in HEK-293A cells. The protein levels of OGT were examined by western blots using anti-OGT antibody and mRNA levels of OGT were determined by RT-PCR from total RNA. We observed that both mRNA and protein levels of OGT were declined when CREB were overexpressed (Figure 5A, 5B). In the contrary, CREB knockdown elevated OGT expression at mRNA and protein level (Figure 5A, 5B). These data suggested that CREB may play a pivotal role in the expression regulation of OGT.

To investigate the molecular mechanisms by which CREB involved in the transcription regulation of OGT, we transfected pGL3/OGT_1500_ alone or together with pCI/HA-CREB into HEK-293A cells and then immunoprecipitated CREB with anti-HA antibody from the cell lysates. The co-immunoprecipitated DNA of OGT promoter with CREB by anti-HA was amplified with PCR by using three sets of primers against different regions (-1446 to -734, -839 to -487, -476 to +32) of OGT promoter respectively (Figure 5C, upper panel). We observed that only -1446 to -734 of the OGT promoter was co-immunoprecipitated with CREB (Figure 5C, lower panel), suggesting that CREB could act on the promoter of OGT.

Domenico Accili et al. demonstrated that CREB is deacetylated by SIRT1 in *SirBACO* mice [20]. It is possible that SIRT1 modulates the expression of OGT dependent on CREB. To verify the speculation, we knocked down CREB by siRNA merely or overexpressed SIRT1 simultaneously in HEK-293A cells which were transfected with pGL3/OGT_1500_. Then the dual luciferase assay was employed to measure luciferase activity. The results showed that knock-down of CREB reversed the inhibition effect of SIRT1 on the luciferase activity driven by OGT promoter (Figure 5D).

To further convince the function of CREB in OGT transcription, we transfected siRNA of CREB alone or together with pcDNA3.1/Myc-SIRT1 into HEK-293A cells. Endogenous OGT was detected by western blot using anti-OGT antibody after 48 h transfection. SIRT1 couldn’t decrease the protein level of OGT when CREB was knocked down (Figure 5E). These results suggested that SIRT1 may act on CREB to negatively regulate the OGT expression.

### SIRT1 physically interacts with CREB

**Figure 6:**
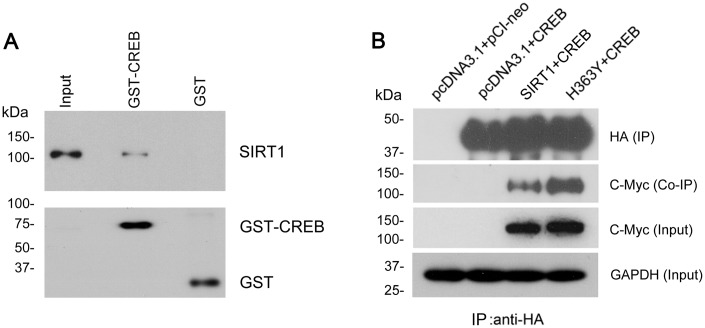
**SIRT1 interacts with CREB.** (**A**) GST-CREB or GST coupled onto glutathione sepharose was incubated with the extract of HEK-293A cells overexpressing SIRT1. After washing, bound proteins were subjected to western blots using anti-GST and anti-SIRT1 antibody. (**B**) SIRT1 could be co-immunoprecipitated by HA-CREB using anti-HA antibody. CREB tagged with HA and SIRT1 tagged with Myc were co-expressed in HEK-293A cells for 48 h. The cell extract was incubated with anti-HA antibody coupled onto protein G beads. The bound proteins were subjected to western blots using anti-HA antibody to CREB and anti-Myc antibody to SIRT1.

To address whether there is interaction between SIRT1 and CREB. GST-pull down was used to detect the protein–protein interaction in *vitro*. We found that SIRT1 overexpressed in HEK-293T cells was pulled down with GST-CREB, but not with GST itself (Figure 6A). To further validate the interaction between SIRT1 and CREB, SIRT1 with Myc-tag and CREB with HA-tag were overexpressed in HEK293A cells and CREB was immunoprecipitated with anti-HA antibody. Then we analyzed whether SIRT1 was co-immunoprecipitated with CREB by western blot using anti-Myc antibody. We detected that Myc-SIRT1 was co-immunoprecipitated with HA-CREB in the immunocomplex (Figure 6B). These data indicate that SIRT1 physically interacted with CREB which is consistent with the results presented by Domenico Accili et al. [20].

### SIRT1 deacetylates CREB and inactivites CREB

**Figure 7:**
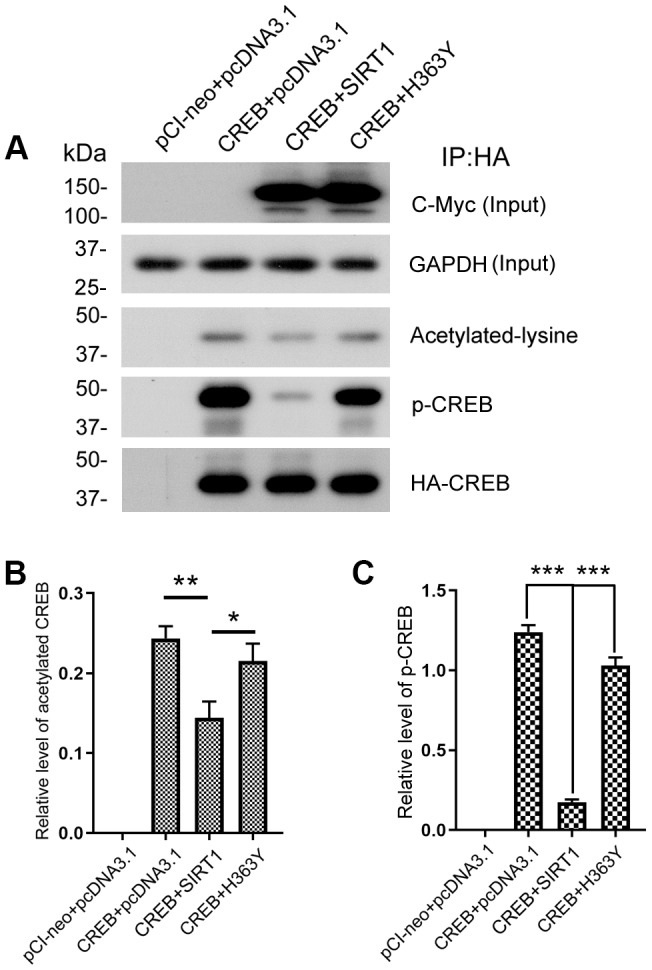
**SIRT1 deacetylates CREB and inhibits phosphorylation of CREB at Ser133.** (**A**) pCI/HA-CREB was co-transfected with pcDNA3.1/SIRT1 or pcDNA3.1/H363Y into HEK-293A cells. CREB was immunoprecipitated with anti-HA and analyzed by western blots developed with anti-CREB or anti-acetylated-lysine or anti-pS133-CREB antibody. The relative levels of CREB acetylation (**B**) or phosphorylation at Ser133 (**C**) are normalized with CREB levels and are represented as mean ± S.D. (n = 3); *, *p* < 0.05, **, *p* < 0.01, ***, *p* < 0.001.

SIRT1 influences the expression of OGT via CREB. To investigate the role of SIRT1 in CREB acetylation, we co-transfected pCI/HA-CREB with pcDNA3.1/Myc-SIRT1 or pcDNA3.1/Myc-H363Y and immunoprecipitated CREB with anti-HA antibody. The acetylation level of immunoprecipitated CREB was analyzed by western blot developed with anti-acetylated-lysine antibody. As shown in Figure 7A and 7B, the level of acetylated CREB was significantly decreased in the cells with overexpression of SIRT1, whereas overexpression of H363Y, the deacetylase-dead mutant of SIRT1, had no effect on the acetylation level of CREB. These results indicate that SIRT1 deacetylates CREB.

A causal link has been established between phosphorylation of CREB at Ser133 and trans-activation of cAMP-responsive genes [32]. Since SIRT1 interacted with and deacetylated CREB, we ask whether SIRT1 affects the activity of CREB. The Immunopurified CREB was also used to test the phosphorylation level of CREB at Ser133 by western blot using anti-pS133-CREB antibody. We found that overexpression of SIRT1 but not H363Y decreased the phosphorylation level of CREB at Ser133 (Figure 7A, 7C), these data are consistent with the findings of Domenico Accili et al. [20].

## DISCUSSION

Elevation of tau O-GlcNAcylation was suggested as pharmacological means to attenuate the formation of tau paired helical filament via decreasing tau phosphorylation [7]. In AD patient’s brain, glucose uptake/metabolism is impaired which contributes to neurodegeneration via decline of O-GlcNAcylation and abnormal hyperphosphorylation of tau [33]. GLUT1 and GLUT3 are the two major brain glucose transporters (GLUT) which are responsible for the transport of glucose from blood into the neuron. Studies also show that the reduction of GLUT1 and GLUT3 is associated with the decrease in O-GlcNAcylation and hyperphosphorylation of tau in AD brain [34]. In the present study, we show for the first time that SIRT1 down-regulates the O-GlcNAcylation of tau by controlling the expression of OGT.

Sirtuins are important in regulating and maintaining glucose and lipid homeostasis [35]. SIRT1, the most well studied member of the mammalian sirtuin family, plays a pivotal role in metabolism, including mitochondrial biogenesis, glycolysis, hypoxia and angiogenesis [36]. SIRT1 is a key regulator in glucose and lipid metabolism by deacetylating certain proteins through its deacetylase activity [37]. We provide the evidence that SIRT1 negatively influence the O-GlcNAcylation level of tau protein via its deacetylase activity. The H363Y form does not alter the mRNA levels of OGT, yet affects the protein levels of OGT (Figure 3). We can’t detect the interaction between SIRT1 and OGT (data not shown). The real reasons need to be investigated further.

Several transcriptional factors involved in transcriptional regulation of key genes in a wide range of metabolic activities are deacetylated by SIRT1, such as nuclear factor-kappa beta (NFκβ) [38], extracellular signal regulated kinase (ERK) [39], the forkhead box subgroup O (FoxO) family [40], peroxisome proliferator-activated receptors γ (PPARγ), and its transcriptional coactivator PPARγ coactivator 1-α (PGC-1α) [41, 42]. CREB, a cellular transcription factor, participates in hepatic gluconeogenesis [43] and lipid synthesis [44]. There are mammalian studies report that the CREB family both activates and represses transcription by a similar mechanism [45]. It has been reported that SIRT1 directly deacetylates CREB at Lys136 to modulate its protein kinase A (PKA)–dependent phosphorylation in transgenic mice overexpressing SIRT1 (SirBACO) [20]. Present study further convinces the interaction between SIRT1 and CREB. Our data also support that SIRT1 deacetylates CREB and decreases phosphorylation level of CREB at Ser133 in cells.

O-GlcNAcylation negatively regulated site-specific phosphorylation of tau [11]. Aberrance of O-GlcNAcylation would result in hyperphosphorylation of tau [35, 46]. In this study, we show that SIRT1 inhibits the O-GlcNAcylation of tau and gives rise to the increased phosphorylation level of tau at Ser199, Ser214 but not Thr212 in which Ser199 is mainly modulated by GSK3β and Ser 214 is reported to be modulated by CDK5 [47]. However, the exact O-GlcNAcylation sites of tau are still not well mapped.

In summary, SIRT1 interacts with and deacetylates CREB, leading to CREB inhibition by reducing the phosphorylation level of CREB at Ser133. Repressed CREB inhibits the expression of OGT, resulting in decreasing O-GlcNAcylation of tau and increasing phosphorylation of tau at specific site. Our results provide a novel insight into molecular mechanisms of the regulation of tau phosphorylation. These findings suggest that SIRT1 may be potential therapeutics target for treating tauopathies.

## MATERIALS AND METHODS

### Primary neuronal cell culture and transduction of viral vectors

Sprague Dawley (SD) rat brains were obtained at embryonic day 18 (E18). The E18 SD rat forebrains were dissected and dissociated in trypsin (0.05% with EDTA) and DNAse (10 μg/mL) and then resuspended in neurobasal medium with 2% B27 and penicillin (20 U/ml) and streptomycin (5 μg/ml) (Gibco, Rockville, MD, USA). For western blots experiments, 6-well plates were seeded with ~1×10^6^ neurons per well. Half of the medium was replaced by fresh medium after 3 days. Then the cerebral cortical neurons were infected with lentiviral-shSIRT1 or its empty vectors (Genepharma, shanghai, China). The virus-containing medium was half-refreshed 24 h later and then subjected for western blot analysis after 72 h.

### Plasmids and antibodies

pGL3-basic, pRL-Tk (thymidine kinase promoter driven Renilla luciferase), and dual luciferase assay kit were bought from Promega (Madison, WI, USA). pCI/GFP-tau_441_ was a gift from Dr. Fei Liu (Department of Neurochemistry, New York State Institute for Basic Research in Developmental Disabilities). shSIRT1 plasmid was purchased from Santa Cruz Biotechnology (Santa Cruz, CA, USA). Mouse monoclonal anti-SIRT1, anti-acetylated-lysine antibody and polyclonal anti-pS133-CREB were from Cell Signaling Technology (Danves, MA, USA). Polyclonal anti-CREB, rabbit polyclonal anti-HA, mouse monoclonal anti-HA and anti-GAPDH antibody were from Sigma (St. Louis, MO, USA). Anti-GFP was a product from Proteintech (Rosemont, IL, USA). RL2 was bought from Affinity BioReagents (Affinity Bioreagents, Golden, CO, USA) and was used for O-GlcNAc detection. Levels of tau phosphorylation at each specific site were determined by using phosphorylation-dependent and site-specific tau antibodies from BioSource International (Camarillo, CA, USA), and total tau level was determined by using R134d, a phosphorylation-independent pan-tau polyclonal antibody produced in laboratory at the New York State Institute for Basic Research in Developmental Disabilities by using the longest human recombinant tau isoform to immune rabbit [48]. Mouse monoclonal anti-Myc, rabbit anti-GAPDH and human siRNA of CREB were purchased from Santa Cruz (Santa Cruz, CA, USA). Peroxidase-conjugated anti-mouse and anti-rabbit IgG were obtained from Jackson Immuno Research Laboratories (West Grove, PA, USA). ECL Kit was from Thermo Scientific (Rockford, IL, USA).

### Plasmid construction

pcDNA3.1/SIRT1 was constructed by subcloning SIRT1 coding region which was PCR amplificated from Flag-SIRT1 plasmid purchased from Addgene (Cambridge, MA, USA) into mammalian expression vector pcDNA3.1 tagged with Myc at C-terminus by BamHI and NotI. Mammalian expression vector pCI/CREB tagged with HA (hemagglutinin) at N-terminus was constructed as described previously [49]. pGEX-4T-1/CREB was constructed by subcloning CREB coding region from pCI/CREB. Luciferase driven by promoter of human *OGT* in pGL3-basic was constructed and confirmed by sequencing.

### Cell culture and transfection

HEK-293A, HEK-293T and COS7 cells were maintained in Dulbecco’s modified Eagle’s medium supplemented with 10% fetal bovine serum (Invitrogen, CA, USA) at 37 °C (5% CO_2_). Transfections were performed with Lipofectamine 2000 (Invitrogen, CA, USA), Lipofectamine 3000 (Invitrogen, CA, USA) or FuGene 6 (Promega, WI, USA), according to the manufacturer’s instructions.

### Reverse-transcription PCR

Total RNA was isolated from cultured cells by using the RNeasy Mini kit (Qiagen, Valencia, CA, USA) and used for first-strand cDNA synthesis with Oligo-(dT)_15–18_ using the RT2 First-Strand Kit (Invitrogen, CA, USA) according to the manufacturer’s instructions. The cDNA of OGT or GAPDH was amplified by PCR using PrimeSTAR HS DNA Polymerase (Takara, Shiga, Japan) at 98 °C for 5 min, 98 °C for 10 s, at 55 °C for 15 s, and at 72 °C for 30 s for 30 cycles and then at 72 °C for 10 min for extension. The PCR products were resolved on 1 % agarose gels and viewed using the Molecular Imager system (Bio-Rad, Hercules, CA, USA). The primers used for this study are as follows: OGT forward 5’- AGG CAG TTC GCT TGT ATC GT-3’ and reverse 5’- TAG AGT AGG CAT CAG CAA AGG T -3’; GAPDH forward 5’- GGT GG T CTC CTC TGA CTT CAA CA -3’ and reverse 5’- GTT GCT GTA GCC AAA TTC GT T GT -3’.

### Plasmid construction of luciferase reporter driven by human tau promoter and luciferase assay

A 1.5-kb fragment of the human OGT genomic DNA from −1463 - +37 bp was amplified by PCR and cloned into pGL3-basic (Promega) by Kpn I and Sma I (New England Biolabs, Ipswich, MA, USA) to generate pGL3/OGT_1500_. The sequence and orientation of the clone was confirmed by DNA sequence analysis. HEK-293A cells were co-transfected with pcDNA3.1, pcDNA3.1/Myc-SIRT1, or pcDNA3.1/Myc-H363Y and pGL3/OGT_1500_, or their control vectors and pRL-Tk for 48 h. The cells were lysed using the passive lysis buffer (Promega). The luciferase activity was measured by the dual luciferase assay kit (Promega) according to manufacturer’s manuals. The firefly luciferase activity and Renilla luciferase activity were measured subsequently and the firefly luciferase activity was normalized with Renilla luciferase activity.

### GST pull down assay

GST and GST-CREB were purified by affinity purification with glutathione sepharose 4B (GE Healthcare, IL, USA). Then the beads was incubated with cell lysates of HEK-293T with Myc-SIRT1 overexpression in buffer (50mM Tris–HCl, pH 7.4, 8.5% sucrose, 50mM NaF, 1mM Na_3_VO_4_, 0.1% Triton X-100, 2mM EDTA, 1mM phenylmethylsulfonyl fluoride, 10 μg/ml aprotinin, 10 μg/ml leupeptin and 10 μg/ml pepstatin) for 4 h at 4 °C. The beads were washed with washing buffer (50mM Tris-HCl, pH 7.4, 150 mM NaCl, 0.1% Triton X-100) six times. Finally, the bound proteins were eluted by boiling in 2× Laemmli sample buffer and the samples were subjected to western blot analysis.

### Co-immunoprecipitation

HEK-293A cells were co-transfected with pCI/HA-CREB and pcDNA3.1/Myc-SIRT1 or pcDNA3.1/Myc-H363Y for 48 h. The cells were washed twice with phosphate-buffered saline (PBS) and lysed by sonication in lysate buffer (50 mM Tris-HCl, pH7.4, 150 mM NaCl, 50 mM NaF, 1 mM Na_3_VO_4_, 2 mM EDTA, 1 mM phenylmethylsulfonyl fluoride, 2 μg/ml aprotinin, 2 μg/ml leupeptin, and 2 μg/ml pepstatin). Insoluble materials were removed by centrifugation at 18,000 g for 10 min. Protein G beads were incubated with mouse anti-HA overnight at 4 °C, and then the antibody bound beads were incubated with the cell lysate at 4 °C for 4 h. The beads were washed with lysate buffer twice and with Tris-buffered saline (TBS) twice, and then bound proteins were eluted by boiling in 2× Laemmli sample buffer for 5 min. The eluted samples were subjected to western blot analysis with the indicated primary antibodies.

### Immuno-affinity purification of tau and CREB

HEK-293A cells were transfected with pCI/GFP-tau_441_ or pCI/HA-CREB for 48 h. HEK-293T cells were transfected with shSIRT1 or control shRNA plasmid (Santa Cruz, CA, USA) together with pCI/GFP-tau_441_ for 48 h and followed by selection with 2 μg/ml puromycin for 72 h. The recombinant GFP-tau or HA-CREB was immunoaffinity-purified from cell extracts in RIPA buffer (50mM Tris–HCl, pH 7.4, 150mM NaCl, 1% NP-40, 2mM EDTA) with monoclonal anti-GFP or anti-HA antibody that was pre-linked covalently onto protein G-agarose beads (Pierce, Rockford, IL). The beads were washed twice each with lysate buffer and with TBS. The bound proteins were eluted by boiling in 2× Laemmli sample buffer. The eluted samples were subjected to western blots with the indicated primary antibodies.

### Chromatin immunoprecipitation

HEK-293A cells co-transfected with pCI/HA-CREB and pGL3/OGT_1500_ were crosslinked with 1% formaldehyde for 10 min at room temperature. After quenching with 125 mM glycine, the cells were lyzed in lysis buffer (16.7 mM Tris–HCl, pH 8.1, 0.01% SDS, 1% Triton X-100, 2 mM EDTA, 167 mM NaCl, 1×Roche protease inhibitors cocktail) on ice for 10 min, and centrifuged at 2000 g for 5 min to pellet nuclei. The nuclear fraction was sonicated in buffer B (50 mM Tris–HCl, pH 8.1, 1% SDS, 10 mM EDTA, 1×protease inhibitors cocktail). After centrifugation at 16 000g for 10 min, the supernatant was subject to immunoprecipitation with anti-HA in IP buffer (16.7 mM Tris–HCl, pH 8.1, 167 mM NaCl, 0.01% SDS, 1% Triton X-100, 2 mM EDTA, 1× protease inhibitors cocktail) for 2 h. Immune-complex was washed sequentially with low-salt buffer (20 mM Tris– HCl, pH 8.1, 150 mM NaCl, 0.1% SDS, 1% Triton X-100 and 2 mM EDTA), with high-salt buffer (20 mM Tris– HCl, pH 8.1, 500 mM NaCl, 0.1% SDS, 1% Triton X-100 and 2 mM EDTA), with LiCl buffer (10 mM Tris–HCl, pH 8.1, 250 mM LiCl, 1% NP-40, 1% deoxycholate and 1 mM EDTA), and with TE buffer (10 mM Tris pH 8.0, 1 mM EDTA). The samples were eluted with 145 μl of 50 mM Tris-HC1 at pH 8.0, 10 mM EDTA, 1% SDS. In total, 120 μl of eluate was decrosslinked at 65 °C for about 7 h and then treated with 1 μg/μl proteinase K for 2 h. Finally, QIAquick PCR Purification kit (Qiagen) was used to purify the immunoprecipitated DNA. PCR reactions were carried out in 30 μl volume with 1/60 of the immunoprecipitated material, PrimeSTAR HS polymerase (TaKaRa) and the corresponding buffer system was used. The following primers were used in the multiplex PCR: forward 1, 5’- GTG ACT CTT GAT TAT GGG ATG GA-3’, reverse 1, 5’- ATG TTG GCC GTA TAG CCT GA -3’; forward 2, 5’- AAC GCC CAG CAT GTC CC-3’, reverse 2, 5’- TGT TAC TTA GCA GGT GGT GTC G -3’; forward 3, 5’- ACA CCA CCT GCT AAG TAA CAA ACA-3’, reverse 3, 5’- CGC CAT CTG GAG CTT CTC G -3’). An initial denaturation for 3 min at 98 °C was followed by 30 cycles with denaturation for 10 sec at 98 °C, annealing for 15 min at 60 °C, polymerization for 1 min at 72 °C and a final extension for 10 min at 72 °C. PCR products were separated on a 1% agarose gel and visualized by ethidium bromide staining.

### Statistical analysis

Where appropriate, the data are presented as the means ± S.D. Data points were compared by the unpaired two-tailed Student’s *t*-test for two groups’ comparison, one-way ANOVA and two-way ANOVA. The calculated *p*-values are indicated in the figures.

## References

[1] Neve RL, Harris P, Kosik KS, Kurnit DM, Donlon TA. Identification of cDNA clones for the human microtubule-associated protein tau and chromosomal localization of the genes for tau and microtubule-associated protein 2. Brain Res. 1986; 387:271–80. 10.1016/0169-328x(86)90033-1 3103857

[2] Mandelkow EM, Mandelkow E. Tau in Alzheimer’s disease. Trends Cell Biol. 1998; 8:425–27. 10.1016/S0962-8924(98)01368-3 9854307

[3] Noble W, Hanger DP, Miller CC, Lovestone S. The importance of tau phosphorylation for neurodegenerative diseases. Front Neurol. 2013; 4:83. 10.3389/fneur.2013.00083 23847585 PMC3696910

[4] Köpke E, Tung YC, Shaikh S, Alonso AC, Iqbal K, Grundke-Iqbal I. Microtubule-associated protein tau. Abnormal phosphorylation of a non-paired helical filament pool in Alzheimer disease. J Biol Chem. 1993; 268:24374–84. 8226987

[5] Park S, Lee JH, Jeon JH, Lee MJ. Degradation or aggregation: the ramifications of post-translational modifications on tau. BMB Rep. 2018; 51:265–73. 10.5483/BMBRep.2018.51.6.077 29661268 PMC6033068

[6] Torres CR, Hart GW. Topography and polypeptide distribution of terminal N-acetylglucosamine residues on the surfaces of intact lymphocytes. Evidence for O-linked GlcNAc. J Biol Chem. 1984; 259:3308–17. 6421821

[7] Arnold CS, Johnson GV, Cole RN, Dong DL, Lee M, Hart GW. The microtubule-associated protein tau is extensively modified with O-linked N-acetylglucosamine. J Biol Chem. 1996; 271:28741–44. 10.1074/jbc.271.46.28741 8910513

[8] Diaferia C, Ghosh M, Sibillano T, Gallo E, Stornaiuolo M, Giannini C, Morelli G, Adler-Abramovich L, Accardo A. Fmoc-FF and hexapeptide-based multicomponent hydrogels as scaffold materials. Soft Matter. 2019; 15:487–96. 10.1039/C8SM02366B 30601569

[9] Gong CX, Liu F, Iqbal K. O-GlcNAcylation: A regulator of tau pathology and neurodegeneration. Alzheimers Dement. 2016; 12:1078–89. 10.1016/j.jalz.2016.02.011 27126545

[10] Hart GW, Greis KD, Dong LY, Blomberg MA, Chou TY, Jiang MS, Roquemore EP, Snow DM, Kreppel LK, Cole RN, Comer FI, Arnold CS, Hayes BK. O-linked N-acetylglucosamine: the “yin-yang” of Ser/Thr phosphorylation? Nuclear and cytoplasmic glycosylation. Adv Exp Med Biol. 1995; 376:115–23. 10.1007/978-1-4615-1885-3_10 8597237

[11] Liu F, Iqbal K, Grundke-Iqbal I, Hart GW, Gong CX. O-GlcNAcylation regulates phosphorylation of tau: a mechanism involved in Alzheimer’s disease. Proc Natl Acad Sci USA. 2004; 101:10804–09. 10.1073/pnas.0400348101 15249677 PMC490015

[12] Liu F, Shi J, Tanimukai H, Gu J, Gu J, Grundke-Iqbal I, Iqbal K, Gong CX. Reduced O-GlcNAcylation links lower brain glucose metabolism and tau pathology in Alzheimer’s disease. Brain. 2009; 132:1820–32. 10.1093/brain/awp099 19451179 PMC2702834

[13] Haltiwanger RS, Holt GD, Hart GW. Enzymatic addition of O-GlcNAc to nuclear and cytoplasmic proteins. Identification of a uridine diphospho-N-acetylglucosamine:peptide beta-N-acetylglucosaminyltransferase. J Biol Chem. 1990; 265:2563–68. 2137449

[14] Braidman I, Carroll M, Dance N, Robinson D, Poenaru L, Weber A, Dreyfus JC, Overdijk B, Hooghwinkel GJ. Characterisation of human N-acetyl-beta-hexosaminidase C. FEBS Lett. 1974; 41:181–84. 10.1016/0014-5793(74)81206-8 4859245

[15] Gao Y, Wells L, Comer FI, Parker GJ, Hart GW. Dynamic O-glycosylation of nuclear and cytosolic proteins: cloning and characterization of a neutral, cytosolic beta-N-acetylglucosaminidase from human brain. J Biol Chem. 2001; 276:9838–45. 10.1074/jbc.M010420200 11148210

[16] Overdijk B, Van der Kroef WM, Van Steijn GJ, Lisman JJ. Isolation and further characterization of bovine brain hexosaminidase C. Biochim Biophys Acta. 1981; 659:255–66. 10.1016/0005-2744(81)90052-8 7260095

[17] Montminy MR, Bilezikjian LM. Binding of a nuclear protein to the cyclic-AMP response element of the somatostatin gene. Nature. 1987; 328:175–78. 10.1038/328175a0 2885756

[18] Alberini CM. Transcription factors in long-term memory and synaptic plasticity. Physiol Rev. 2009; 89:121–45. 10.1152/physrev.00017.2008 19126756 PMC3883056

[19] Teich AF, Nicholls RE, Puzzo D, Fiorito J, Purgatorio R, Fa’ M, Arancio O. Synaptic therapy in Alzheimer’s disease: a CREB-centric approach. Neurotherapeutics. 2015; 12:29–41. 10.1007/s13311-014-0327-5 25575647 PMC4322064

[20] Qiang L, Lin HV, Kim-Muller JY, Welch CL, Gu W, Accili D. Proatherogenic abnormalities of lipid metabolism in SirT1 transgenic mice are mediated through Creb deacetylation. Cell Metab. 2011; 14:758–67. 10.1016/j.cmet.2011.10.007 22078933 PMC3237922

[21] Anderson KA, Green MF, Huynh FK, Wagner GR, Hirschey MD. SnapShot: Mammalian Sirtuins. Cell. 2014; 159:956–956.e1. 10.1016/j.cell.2014.10.045 25417168 PMC4337867

[22] Michishita E, Park JY, Burneskis JM, Barrett JC, Horikawa I. Evolutionarily conserved and nonconserved cellular localizations and functions of human SIRT proteins. Mol Biol Cell. 2005; 16:4623–35. 10.1091/mbc.e05-01-0033 16079181 PMC1237069

[23] Zhang F, Wang S, Gan L, Vosler PS, Gao Y, Zigmond MJ, Chen J. Protective effects and mechanisms of sirtuins in the nervous system. Prog Neurobiol. 2011; 95:373–95. 10.1016/j.pneurobio.2011.09.001 21930182 PMC3242010

[24] Chalkiadaki A, Guarente L. High-fat diet triggers inflammation-induced cleavage of SIRT1 in adipose tissue to promote metabolic dysfunction. Cell Metab. 2012; 16:180–88. 10.1016/j.cmet.2012.07.003 22883230 PMC3539750

[25] Brunet A, Sweeney LB, Sturgill JF, Chua KF, Greer PL, Lin Y, Tran H, Ross SE, Mostoslavsky R, Cohen HY, Hu LS, Cheng HL, Jedrychowski MP, et al. Stress-dependent regulation of FOXO transcription factors by the SIRT1 deacetylase. Science. 2004; 303:2011–15. 10.1126/science.1094637 14976264

[26] Huang H, Tindall DJ. Dynamic FoxO transcription factors. J Cell Sci. 2007; 120:2479–87. 10.1242/jcs.001222 17646672

[27] Chen J, Zhou Y, Mueller-Steiner S, Chen LF, Kwon H, Yi S, Mucke L, Gan L. SIRT1 protects against microglia-dependent amyloid-beta toxicity through inhibiting NF-kappaB signaling. J Biol Chem. 2005; 280:40364–74. 10.1074/jbc.M509329200 16183991

[28] Yeung F, Hoberg JE, Ramsey CS, Keller MD, Jones DR, Frye RA, Mayo MW. Modulation of NF-kappaB-dependent transcription and cell survival by the SIRT1 deacetylase. EMBO J. 2004; 23:2369–80. 10.1038/sj.emboj.7600244 15152190 PMC423286

[29] Kreppel LK, Hart GW. Regulation of a cytosolic and nuclear O-GlcNAc transferase. Role of the tetratricopeptide repeats. J Biol Chem. 1999; 274:32015–22. 10.1074/jbc.274.45.32015 10542233

[30] Cartharius K, Frech K, Grote K, Klocke B, Haltmeier M, Klingenhoff A, Frisch M, Bayerlein M, Werner T. MatInspector and beyond: promoter analysis based on transcription factor binding sites. Bioinformatics. 2005; 21:2933–42. 10.1093/bioinformatics/bti473 15860560

[31] Quandt K, Frech K, Karas H, Wingender E, Werner T. MatInd and MatInspector: new fast and versatile tools for detection of consensus matches in nucleotide sequence data. Nucleic Acids Res. 1995; 23:4878–84. 10.1093/nar/23.23.4878 8532532 PMC307478

[32] Gonzalez GA, Montminy MR. Cyclic AMP stimulates somatostatin gene transcription by phosphorylation of CREB at serine 133. Cell. 1989; 59:675–80. 10.1016/0092-8674(89)90013-5 2573431

[33] Gong CX, Liu F, Grundke-Iqbal I, Iqbal K. Impaired brain glucose metabolism leads to Alzheimer neurofibrillary degeneration through a decrease in tau O-GlcNAcylation. J Alzheimers Dis. 2006; 9:1–12. 10.3233/JAD-2006-9101 16627930

[34] Liu Y, Liu F, Iqbal K, Grundke-Iqbal I, Gong CX. Decreased glucose transporters correlate to abnormal hyperphosphorylation of tau in Alzheimer disease. FEBS Lett. 2008; 582:359–64. 10.1016/j.febslet.2007.12.035 18174027 PMC2247364

[35] Houtkooper RH, Pirinen E, Auwerx J. Sirtuins as regulators of metabolism and healthspan. Nat Rev Mol Cell Biol. 2012; 13:225–38. 10.1038/nrm3293 22395773 PMC4872805

[36] Knight JR, Milner J. SIRT1, metabolism and cancer. Curr Opin Oncol. 2012; 24:68–75. 10.1097/CCO.0b013e32834d813b 22080944

[37] Ye X, Li M, Hou T, Gao T, Zhu WG, Yang Y. Sirtuins in glucose and lipid metabolism. Oncotarget. 2017; 8:1845–59. 10.18632/oncotarget.12157 27659520 PMC5352102

[38] Salminen A, Huuskonen J, Ojala J, Kauppinen A, Kaarniranta K, Suuronen T. Activation of innate immunity system during aging: NF-kB signaling is the molecular culprit of inflamm-aging. Ageing Res Rev. 2008; 7:83–105. 10.1016/j.arr.2007.09.002 17964225

[39] Du LL, Xie JZ, Cheng XS, Li XH, Kong FL, Jiang X, Ma ZW, Wang JZ, Chen C, Zhou XW. Activation of sirtuin 1 attenuates cerebral ventricular streptozotocin-induced tau hyperphosphorylation and cognitive injuries in rat hippocampi. Age (Dordr). 2014; 36:613–23. 10.1007/s11357-013-9592-1 24142524 PMC4039268

[40] Kitamura YI, Kitamura T, Kruse JP, Raum JC, Stein R, Gu W, Accili D. FoxO1 protects against pancreatic beta cell failure through NeuroD and MafA induction. Cell Metab. 2005; 2:153–63. 10.1016/j.cmet.2005.08.004 16154098

[41] Rodgers JT, Lerin C, Haas W, Gygi SP, Spiegelman BM, Puigserver P. Nutrient control of glucose homeostasis through a complex of PGC-1alpha and SIRT1. Nature. 2005; 434:113–18. 10.1038/nature03354 15744310

[42] Saunders LR, Sharma AD, Tawney J, Nakagawa M, Okita K, Yamanaka S, Willenbring H, Verdin E. miRNAs regulate SIRT1 expression during mouse embryonic stem cell differentiation and in adult mouse tissues. Aging (Albany NY). 2010; 2:415–31. 10.18632/aging.100176 20634564 PMC2933889

[43] Chrivia JC, Kwok RP, Lamb N, Hagiwara M, Montminy MR, Goodman RH. Phosphorylated CREB binds specifically to the nuclear protein CBP. Nature. 1993; 365:855–59. 10.1038/365855a0 8413673

[44] Herzig S, Hedrick S, Morantte I, Koo SH, Galimi F, Montminy M. CREB controls hepatic lipid metabolism through nuclear hormone receptor PPAR-gamma. Nature. 2003; 426:190–93. 10.1038/nature02110 14614508

[45] Mirisis AA, Alexandrescu A, Carew TJ, Kopec AM. The Contribution of Spatial and Temporal Molecular Networks in the Induction of Long-term Memory and Its Underlying Synaptic Plasticity. AIMS Neurosci. 2016; 3:356–84. 10.3934/Neuroscience.2016.3.356 27819030 PMC5096789

[46] Deng Y, Li B, Liu Y, Iqbal K, Grundke-Iqbal I, Gong CX. Dysregulation of insulin signaling, glucose transporters, O-GlcNAcylation, and phosphorylation of tau and neurofilaments in the brain: implication for Alzheimer’s disease. Am J Pathol. 2009; 175:2089–98. 10.2353/ajpath.2009.090157 19815707 PMC2774072

[47] Liu F, Iqbal K, Grundke-Iqbal I, Gong CX. Involvement of aberrant glycosylation in phosphorylation of tau by cdk5 and GSK-3beta. FEBS Lett. 2002; 530:209–14. 10.1016/S0014-5793(02)03487-7 12387894

[48] Gong CX, Lidsky T, Wegiel J, Zuck L, Grundke-Iqbal I, Iqbal K. Phosphorylation of microtubule-associated protein tau is regulated by protein phosphatase 2A in mammalian brain. Implications for neurofibrillary degeneration in Alzheimer’s disease. J Biol Chem. 2000; 275:5535–44. 10.1074/jbc.275.8.5535 10681533

[49] Liu H, Jin X, Yin X, Jin N, Liu F, Qian W. PKA-CREB Signaling Suppresses Tau Transcription. J Alzheimers Dis. 2015; 46:239–48. 10.3233/JAD-142610 25720403

